# Knockdown of *CYP6SZ3* and *CYP6AEL1* genes increases the susceptibility of *Lasioderma serricorne* to ethyl formate and benzothiazole

**DOI:** 10.3389/fphys.2024.1503953

**Published:** 2024-11-20

**Authors:** Xiaokun Li, Lixin Ma, Wenjia Yang, Kangkang Xu

**Affiliations:** Key Laboratory of Surveillance and Management of Invasive Alien Species in Guizhou Education Department, Guiyang University, Guiyang, China

**Keywords:** *Lasioderma serricorne*, cytochrome P450, gene expression, detoxification, molecular docking

## Abstract

Insect cytochrome P450 monooxygenases (CYPs) play crucial roles in the metabolic detoxification of insecticides. Ethyl formate and benzothiazole have recently regained popularity as fumigants due to rising resistance to phosphine in the stored-product pests. However, the mechanisms underlying tolerance to these two fumigants in *Lasioderma serricorne*, a major global insect pest of stored products, remain poorly understood. In this study, two *CYP* genes, named *CYP6SZ3* and *CYP6AEL1*, were identified from *L. serricorne*, belonging to the CYP6 family and containing five conserved domains characteristic of CYP proteins. Spatiotemporal expression analysis revealed that both genes were predominantly expressed in the larval stage and showed the highest expression in the foregut. Upon exposure to ethyl formate and benzothiazole, both genes were upregulated, with significantly increased transcription levels following treatment. RNA interference-mediated silencing of *CYP6SZ3* and *CYP6AEL1* led to increased susceptibility and significantly higher mortality of *L. serricorne* when exposed to these fumigants. Homology modeling and molecular docking analyses showed stable binding of these fumigants to CYP6SZ3 and CYP6AEL1 proteins, with binding free energies from −26.88 to −94.68 kcal mol^−1^. These findings suggest that the induction of *CYP6SZ3* and *CYP6AEL1* is likely involved in the detoxification of ethyl formate and benzothiazole in *L. serricorne*.

## 1 Introduction

Insect cytochrome P450 monooxygenases (CYPs or P450s), encoded by a highly conserved superfamily of genes, are involved in the oxidative metabolism of both endogenous compounds (like fatty acids and hormones) and exogenous substrates (including chemical insecticides and plant allelochemicals) (Katsavou et al., 2022; Li et al., 2007; Schuler, 2011). Insect P450 enzymes typically consist of approximately 500 amino acids and possess a characteristic heme-binding domain with the FxxGxRxCxG recognition motif (Feyereisen and Gilbert, 2012). Although the number of CYP genes varies significantly across insect species, they are classified into six primary clans based on phylogenetic relationships: CYP2, CYP3, CYP4, mitochondrial CYP, and the CYP20 and CYP16 clans (Dermauw et al., 2020). Substantial evidence highlights the roles of the CYP3 clan, particularly members of the CYP6 and CYP9 families, in xenobiotic metabolism and direct detoxification processes (Lu et al., 2021). Numerous CYPs involved in insecticide detoxification, predominantly from the CYP6 and CYP9 families, have been identified and functionally characterized. For example, RNA interference (RNAi)-mediated silencing of genes such as *CYP6DW4*, *CYP6DW5*, *CYP6DW6*, and *CYP6DZ8* greatly increased mortality of *Bemisia tabaci* to imidacloprid (Qin et al., 2023). Reducing the expression of *CYP9A9* significantly increased the susceptibility of *Spodoptera exigua* to lufenuron and methoxyfenozide (Zhang L. J. et al., 2023). Similarly, knockdown of *CYP6BQ7* in *Tribolium castaneum* reduced tolerance to essential oil of *Artemisia vulgaris*, linking this gene to plant toxicant detoxification (Zhang et al., 2021). In *Helicoverpa armigera*, knocking out the *CYP6AE* cluster by genome editing dramatically decreased survival rate of the insects when exposed to different insecticides and phytochemical toxins (Wang et al., 2018). Heterologous expression studies confirmed the enzymatic activities of various CYP6 and CYP9 proteins, such as *Helicoverpa armigera CYP9A12* and *CYP9A14*, which can metabolize pyrethroid (Yang et al., 2008), *Laodelphax striatellus CYP6AY3v2*, which can metabolize imidacloprid and indoxacarb (Wang et al., 2017), *Chilo suppressalis CYP6CV5*, which metabolizes imidacloprid and chlorantraniliprole (Xu et al., 2024), and *Meligethes aeneus CYP6BQ23*, which metabolizes deltamethrin (Zimmer et al., 2014), *Cydia pomonella CYP9A120* and *CYP9A121*, which metabolize lambda-cyhalothrin (Li et al., 2023). Functional expression of *Ceratitis capitata CYP6A51* in transgenic *Drosophila melanogaster* demonstrated a significant decrease in pyrethroid tolerance, supported by *in vitro* metabolism assays (Tsakireli et al., 2019). These studies underscore the crucial role of *CYP6* and *CYP9* genes in insecticide detoxification.

Chemical fumigation remains a common and effective strategy for controlling stored insect pests, relying on high concentrations of gaseous agents to target pests (Austel et al., 2017). Widely used fumigants include phosphine (Nayak et al., 2020), sulfuryl fluoride, methyl bromide, carbonyl sulfide (Aribou and Ng, 2022), and allyl isothiocyanate (Zhang et al., 2017). However, the extensive use of these limited options has led to the development of fumigant resistance in insects, environmental pollution (Boyer et al., 2012), and regulatory restrictions on some fumigants, creating an urgent need for alternative approaches. Ethyl formate, a naturally occurring compound found in the atmosphere, soil, water, vegetation, and various foods (e.g., beer, wine, grapes, wheat, barley, raisin, rice, and cheese) (Zaitoon et al., 2021; Ren and Mahon, 2006), degrades into ethanol and formic acid upon air exposure (Haritos and Dojchinov, 2003). Ethyl formate is increasingly being evaluated as a fumigant against stored pests such *Scirtothrips dorsalis* (Kim et al., 2023), *Macchiademus diplopterus* (Smit et al., 2020), *Drosophila suzukii* (Zaitoon et al., 2021), and *Sitophilus oryzae* (Damcevski et al., 2010), with minimal toxicity to plants, crops, and food. Benzothiazole compounds possess diverse biological activities, including antimicrobial, anticancer, antifungal, anthelmintic, antileishmanial, and anticonvulsant properties (Mithlesh et al., 2010). Benzothiazole demonstrates potent ovicidal, larvicidal, pupicidal, and adulticidal effects against *T*. *castaneum* and disrupts normal feeding and digestion in *Bradysia odoriphaga* (Cui et al., 2020a; Cui et al., 2020b; Zhao et al., 2016a). Sublethal concentrations of benzothiazole prolonged the developmental periods of eggs, larvae, pupae of *B. odoriphaga*, and decreased the survival rate and female fecundity (Zhao et al., 2016b).

The cigarette beetle, *Lasioderma serricorne* (Fabricius) (Coleoptera: Anobiidae), was first discovered in dried resin within the tomb of Tutankhamun (Maille et al., 2023). The larvae primarily feed on stored tobacco, grains, and other products, causing substantial economic losses (Baliota et al., 2023). Currently, the management of *L. serricorne* relies heavily on phosphine fumigation, which is becoming less effective due to the emergence of a resistant population (Edde, 2019; Sağlam et al., 2015). Ethyl formate fumigation has demonstrated high toxicity against *L. serricorne* eggs, pupae, and adults, suggesting a promising alternative for pest control (Kim et al., 2020). However, the detoxification mechanisms of *L. serricorne* for ethyl formate and benzothiazole remain poorly understood. Insights into P450-mediated detoxification pathways could aid in developing more effective pest management strategies. This study aimed to: (1) characterize the full-length open reading frames (ORFs) of two *CYP6* genes in *L. serricorne*; (2) examine the temporal and spatial expression profiles of *CYP6SZ3* and *CYP6AEL1*; (3) assess the transcriptional response of these genes to ethyl formate and benzothiazole exposure; (4) evaluate the roles of *CYP6SZ3* and *CYP6AEL1* in the detoxification of these fumigants; and (5) perform homology modeling and molecular docking analyses of CYP6SZ3 and CYP6AEL1 interactions with the fumigants.

## 2 Materials and methods

### 2.1 Insect rearing and chemicals

A population of *L. serricorne* was originally collected from Guiyang City, Guizhou Province, China (Yang et al., 2019). Larvae were reared in dried roots of *Angelica sinensis* at 28°C ± 1°C, with 40% ± 10% relative humidity, and kept in continuous darkness in a laboratory without insecticide exposure. Ethyl formate (≥98% purity) was obtained from Shanghai Aladdin Biochemical Technology Co., Ltd., Shanghai, China, while benzothiazole (>96% purity) was supplied by the TCI (Shanghai) Development Co., Ltd., Shanghai, China.

### 2.2 Toxicity bioassay

Toxicity tests were conducted using a sealed bottle method as described previously (Yan et al., 2020). Ethyl formate or benzothiazole was applied to a 1 × l cm filter paper strip attached near the cap of a wide-mouthed bottle. Fourth-instar larvae were then placed in the sealed bottles and exposed to the fumigants. A control group of larvae was kept without fumigant exposure. Mortality was recorded after 24 h, with immobile individuals considered dead. Each bioassay involved five fumigant concentrations and a control group, with three to eight replicates for each treatment. Thirty larvae were randomly selected for each replicate. The sublethal concentration (LC_30_) values, median lethal concentration (LC_50_) values, and 95% confidence intervals were determined using standard probit analysis in SPSS 20.0 software (SPSS Inc., Chicago, IL, United States).

### 2.3 Gene cloning and bioinformatic analysis

Two *CYP6* cDNA sequences were identified from the *L. serricorne* transcriptome (SRR13065789) and confirmed by reverse-transcription polymerase chain reaction (RT-PCR) using gene-specific primers (Supplementary Table S1). The nomenclatures of two *CYP6* genes (*CYP6SZ3* and *CYP6AEL1*) were provided by Dr. David R. Nelson from the Cytochrome P450 Nomenclature Committee. Molecular weight and isoelectric points (pI) were analyzed using ExPASy (https://web.expasy.org/protparam/). Multiple sequence alignment was conducted with CLC Sequence Viewer 8 (Qiagen, Aarhus A/S, www.qiagenbioinformatics.com), and conserved motifs were predicted using GENEDOC. The phylogenetic analysis was performed using the protein sequences from the CYP3 clan of Coleoptera insects obtained from the National Center for Biotechnology Information. A phylogenetic tree was constructed by MEGA 7.0 software using the neighbor-joining method with 1,000 bootstrap replicates (Kumar et al., 2016).

### 2.4 Fumigant treatment and sample collection

The fourth-instar larvae were treated with the LC_30_ and LC_50_ concentrations of ethyl formate and benzothiazole using the sealed method as described above. The surviving larvae were collected at 24 h after fumigants treatment. For each treatment, thirty individuals were used, with three replicates per treatment. Different developmental stages, including 1-day-old eggs, first-to fourth-instar larvae, prepupae, pupae, and adults, were collected for temporal expression analysis. For spatial expression analysis, specific tissues from fourth-instar larvae (epidermis, Malpighian tubules, fat body, foregut, midgut, and hindgut) were collected. The larvae were dissected on RNAhold® Reagent (TransGen Biotech, Beijing, China) under stereomicroscope (Olympus, Tokyo, Japan) to maintain tissue freshness. Each sample had three replicates, with 30–50 individuals per replicate.

### 2.5 RNA extraction and cDNA synthesis

Total RNA was extracted from whole bodies or specific tissue using the TransZol reagent (TransGen Biotech, Beijing, China). RNA quality and purity were assessed with a NanoDrop1000 spectrophotometer (Thermo Scientific, Waltham, MA, United States) and further confirmed by agarose gel electrophoresis. First-strand cDNA was synthesized using the TransScript Synthesis Supermix (TransGen Biotech) with 1 μg of total RNA.

### 2.6 Real-time quantitative PCR (qPCR)

qPCR was performed using a CFX-96 real-time PCR system (Bio-Rad, Hercules, CA, United States) with TransStart® Green qPCR Supermix (TransGen Biotech), following the manufacturer’s protocol. PCR amplification was carried out with the following conditions: 95°C for 5 min, followed by 40 cycles of 95°C for 15 s and 60°C for 30 s. A melting curve analysis was performed from 55°C to 95°C to verify primer specificity. The gene expression level was calculated using the 2^−ΔΔCT^ method (Livak and Schmittgen, 2001) and normalized to the reference genes *elongation factor 1-alpha* (*EF1α*) and *18S ribosomal RNA* (*18S*) (Yang et al., 2020). All the qPCR primers are presented in Supplementary Table S1.

### 2.7 RNA interference (RNAi) and bioassay

The cDNA fragments of *CYP6SZ3*, *CYP6AEL1*, and *green fluorescent protein* (*GFP*, as a negative control) were used to design double-stranded RNA (dsRNA) primers (Supplementary Table S1) through E-RNAi (https://www.dkfz.de/signaling/e-rnai3/). These dsRNAs were synthesized *in vitro* using the TranscriptAid T7 High Yield Transcription Kit (Thermo Scientific, Wilmington, DE, United States). Each fourth-instar larva was injected with 200 ng dsRNA (1 μg/μL) of *CYP6SZ3*, *CYP6AEL1*, or *GFP* using a Nanoliter 2010 injector (World Precision Instruments, Sarasota, FL, United States). At 48 h after injection, whole-body samples were collected to assess RNAi efficiency. In a parallel experiment, surviving larvae were collected at 48 h after dsRNA injection and further treated with ethyl formate and benzothiazole at LC_30_ and LC_50_ concentrations, and the mortality was recorded 24 h later. Each treatment was conducted in four replicates, with 30 larvae per replicate.

### 2.8 Homology modeling and molecular docking

Three-dimensional models of ethyl formate and benzothiazole were obtained from PubChem (https://pubchem.ncbi.nlm.nih.gov/). The protein sequences of CYP6SZ3 and CYP6AEL1 were modeled using Swiss-Model server (https://swissmodel.expasy.org/). Discovery Studio Client v21.1 (Accelrys Inc., San Diego, CA, United States) was used to identify potential fumigant ligand-binding pockets in the modeled CYP structures with default parameters. Binding free energies (∆G_bind_) between CYP proteins and fumigant ligands were calculated, and the outcomes were visualized using PyMOL (Delano Scientific, San Carlos, CA, United States).

### 2.9 Data analysis

Data are represented as the means ± standard error. Statistical differences were determined by one-way analysis of variance (ANOVA), followed by a least significant difference test or Student’s *t*-test using SPSS 20.0 software.

## 3 Results

### 3.1 Toxicity of fumigants against *L. serricorne* larvae

The toxicity of two fumigants was evaluated on fourth-instar larvae using the sealed fumigation method with wide-mouth bottles. The LC_30_ values for ethyl formate and benzothiazole were determined to be 19.344 and 396.751 μL L^−1^, respectively. The LC_50_ values were 21.621 μL L^−1^ for ethyl formate and 725.616 μL L^−1^ for benzothiazole (Table 1).

**TABLE 1:** Toxicity of two fumigants against *L. serricorne*.

Fumigants	Slope ± SE	LC_30_ (95% CL) (μL L^−1^)	LC_50_ (95% CL) (μL L^−1^)	X^2^	*P*	df
Ethyl formate	10.850 ± 1.172	19.344 (18.522, 19.976)	21.621 (21.038, 22.202)	4.752	0.98025	13
Benzosulfonazole	2.00 0 ± 0.086	396.751 (322.406, 456.231)	725.616 (652.747, 797.332)	98.238	0.00001	36

CL, confidence limits.

### 3.2 Characterization of two CYP6 genes from *L. serricorne*

**FIGURE 1:**
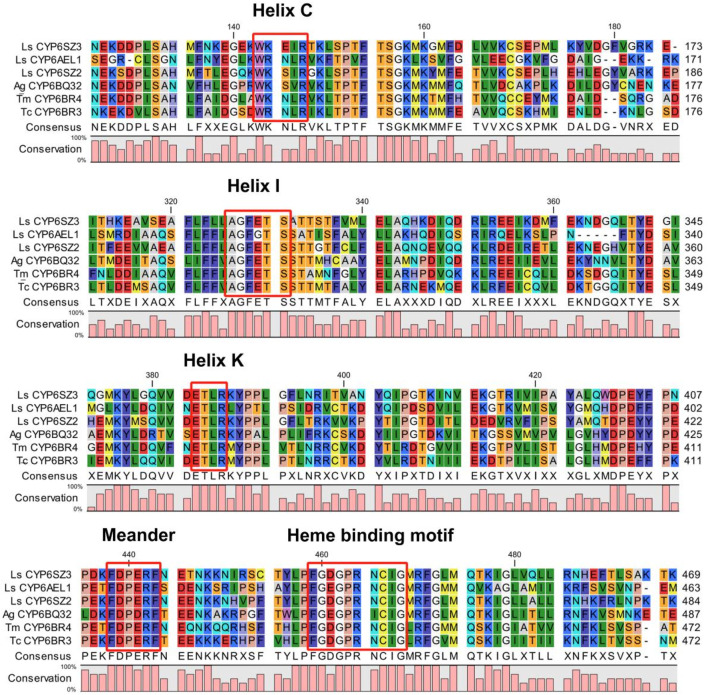
Alignment of amino acid sequences of six insect CYP6 proteins. Five conserved motifs are highlighted in red boxes. Insect species included *Anoplophora glabripennis* (Ag), *Tribolium castaneum* (Tc), *Tenebrio molitor* (Tm), and *Lasioderma serricorne* (Ls).

**FIGURE 2:**
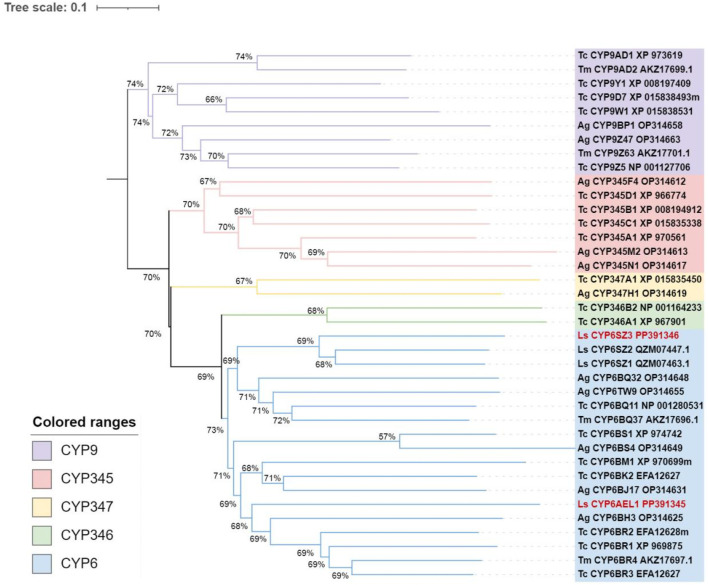
Phylogenetic analysis of CYP3 clan proteins in Coleopteran insects. The tree was constructed using the neighbor-joining method with 1,000 bootstrap replicates. The sequences of *L. serricorne* CYP6SZ3 and CYP6AEL1 are highlighted in red font. Insect species included *Anoplophora glabripennis* (Ag), *Tribolium castaneum* (Tc), *Tenebrio molitor* (Tm), and *Lasioderma serricorne* (Ls). GenBank accession numbers for all sequences are provided in the tree.

Two candidate *CYP6* genes were identified from *L. serricorne*, the full-length ORFs of *CYP6SZ1* and *CYP6AEL1* were verified by RT-PCR and named by David R. Nelson*.* The ORF sequences of *CYP6SZ1* and *CYP6AEL1* were 1,485 bp and 1,977 bp, encoding 494 and 656 amino acid residues, with predicted molecular weights of 56.69 kDa and 74.71 kDa and pI of 8.85 and 9.03, respectively. Sequence alignment analyses revealed that CYP6SZ1 and CYP6AEL1 contained five conserved motifs, including the helix C (WxxxR), helix I (AGxxTS), helix K (ExLR), meander (FxPxxF), and heme binding (FxxGxxxCxG) (Figure 1). Phylogenetic analysis showed that both CYP6SZ3 and CYP6AEL1 belonged to the CYP3 clan from other insect species. The results showed that these CYPs clustered in five distinct families, and CYP6SZ3 and CYP6AEL1 belonged to the CYP6 family (Figure 2), with CYP6SZ3 closely related to CYP6SZ2 and CYP6SZ1 of *L. serricorne*, and CYP6AEL1 to CYP6BH3 of *Anoplophora glabripennis*.

### 3.3 Developmental and tissue-specific expression analysis

**FIGURE 3:**
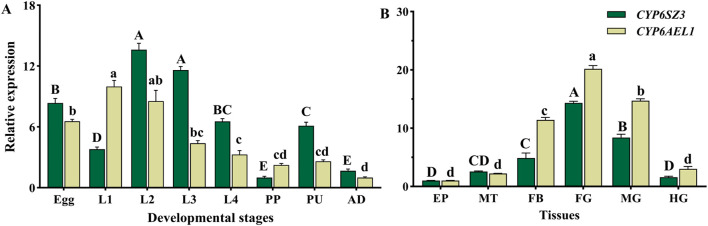
Relative expression levels of *CYP6SZ3* and *CYP6AEL1* across different developmental stages **(A)** and tissues **(B)** in *L. serricorne*. Stages: L1 (first-instar larvae), L2 (second-instar larvae), L3 (third-instar larvae), L4 (fourth-instar larvae), PP (prepupae), PU (pupae), and AD (adults). Tissues: EP (epidermis), MT (Malpighian tubules), FB (fat body), FG (foregut), MG (midgut), and HG (hindgut). Uppercase letters indicate significant differences in *CYP6SZ3* expression, and lowercase letters indicate significant differences in *CYP6AEL1 e*xpression, based on one-way ANOVA followed by a least significant difference test (*P* < 0.05).

*CYP6SZ3* and *CYP6AEL1* were expressed across all developmental stages of *L. serricorne*, from eggs to adults. The highest expression of *CYP6SZ3* was observed in the second-instar larvae, 13.6-fold higher than in the prepupa, which had the lowest expression level. *CYP6AEL1* showed the highest expression in the first-instar larvae, 9.9-fold higher than in adults (Figure 3A). *CYP6SZ3* and *CYP6AEL1* exhibited similar tissue-specific expression patterns, and both genes were mainly expressed in the foregut, midgut, and fat body, with the highest expression levels in the foregut,14.3-fold for *CYP6SZ3* and 20.2-fold for *CYP6AEL1* compared to the epidermis (Figure 3B).

### 3.4 Expression patterns of two *CYP6* genes in response to fumigants

**FIGURE 4:**
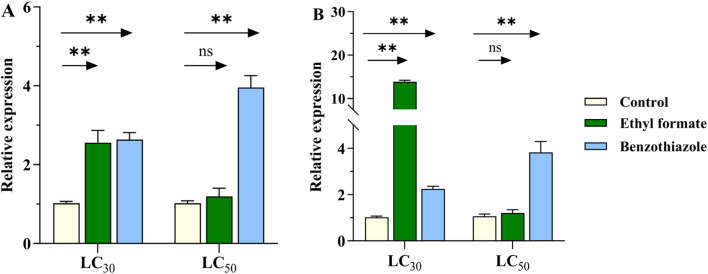
Effect of fumigant treatments on the expression levels of *CYP6SZ3* **(A)** and *CYP6AEL1* **(B)** in *L. serricorne*. Larvae exposed to normal air served as the negative control. Significant differences between the treatments and control at the same concentration were assessed using Student’s *t*-test (**P < 0.01).

The expression of *CYP6SZ3* and *CYP6AEL1* increased significantly after treatment with LC_30_ of ethyl formate, showing 2.6- and 13.8-fold increases, respectively. However, no significant change was observed at LC_50_ concentrations. For benzothiazole exposure, expression of *CYP6SZ3* increased by 2.6- and 3.9-fold at LC_30_ and LC_50_ concentrations, while *CYP6AEL1* showed 2.2- and 3.8-fold increases compared to the control (Figure 4).

### 3.5 Knockdown of *CYP6SZ3* and *CYP6AEL1* by RNAi

**FIGURE 5:**
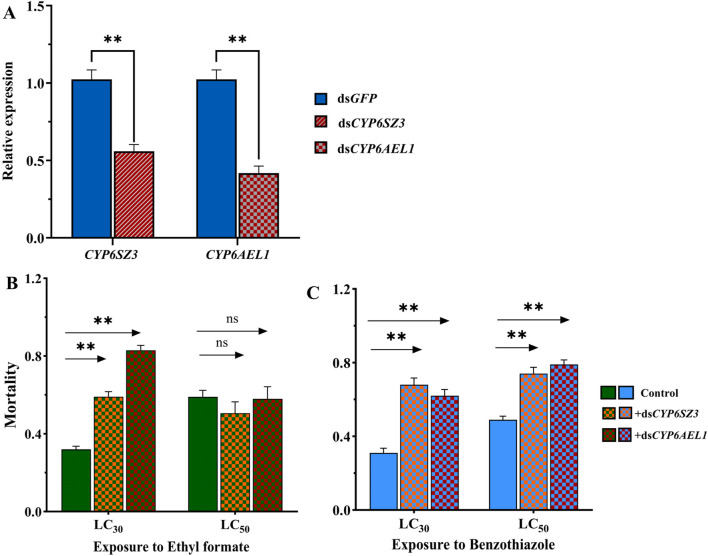
Functional analysis of *CYP6SZ3* and *CYP6AEL1* by RNAi. **(A)** Relative expression levels of *CYP6SZ3* and *CYP6AEL1* in larvae at 48 h after dsRNA injection. Mortality rates of dsRNA-injected larvae treated with LC_30_ and LC_50_ of ethyl formate **(B)** and benzothiazole **(C)**. Significant differences between the treatments and control were determined by Student’s *t*-test (**P < 0.01).

RNAi was performed in *L. serricorne* larvae to analyze the functions of *CYP6SZ3* and *CYP6AEL1* in fumigant detoxification. At 48 h after dsRNA injection, the expression levels of *CYP6SZ3* and *CYP6AEL1* were significantly decreased by 44.1% and 58.2% compared with the control, respectively (Figure 5A). The subsequent bioassay results showed that silencing both genes significantly increased susceptibility to LC_30_ of ethyl formate, with mortalities rising by 84.66% for *CYP6SZ3* and 159.37% for *CYP6AEL1*. No significant difference in mortality was observed after LC_50_ exposure to ethyl formate (Figure 5B). In contrast, after exposure to LC_30_ and LC_50_ of benzothiazole, mortality increased by 119.35% and 100% in the ds*CYP6SZ3* group and by 51.02% and 61.22% in the ds*CYP6AEL1* group (Figure 5C).

### 3.6 Homology modeling and fumigant docking

**FIGURE 6:**
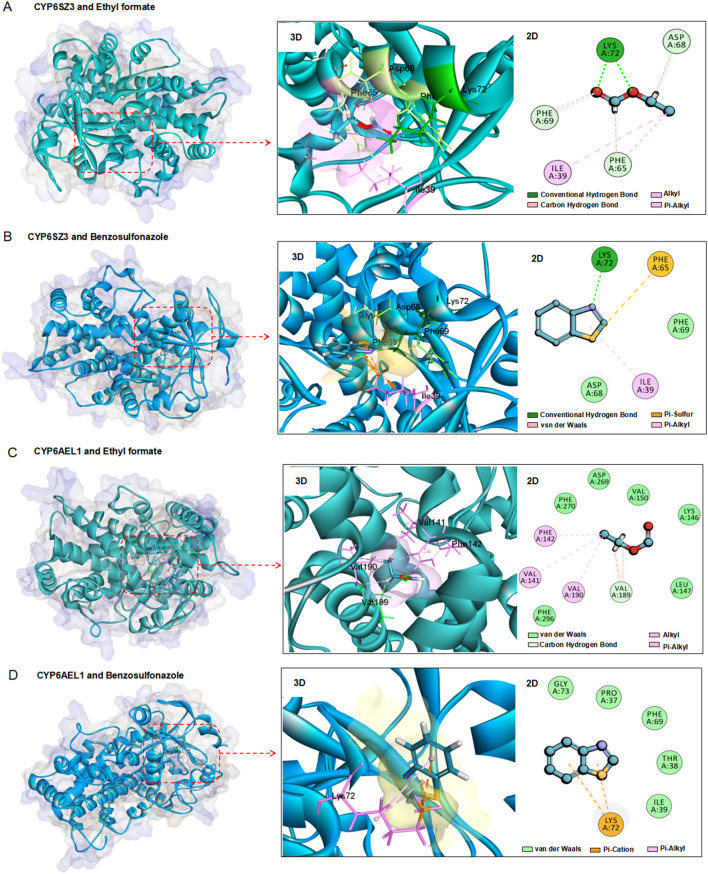
Homology modeling and fumigant docking. **(A, B)** Three-dimensional structures of CYP6SZ3 with two fumigants. **(C, D)** Three-dimensional structures of CYP6AEL1 with two fumigants. Amino acids within a distance of 6.0 Å from the docked ethyl formate or benzothiazole are enclosed in red boxes. Ethyl formate and benzothiazole are depicted as sticks, with oxygen in red, carbon in blue, sulfur in yellow, and nitrogen in purple.

The three-dimensional structures of the CYP6SZ3 and CYP6AEL1 proteins were predicted using the Swiss-Model Server. Molecular docking was performed to investigate the binding interactions of these two CYP6 proteins with ethyl formate and benzothiazole. The docking models revealed strong interactions between both proteins and the compounds. For CYP6SZ3, the predicted binding site for ethyl formate included residues Lys72, Phe69, Asp68, Phe65, and Ile39, forming hydrogen bonds, carbon-hydrogen bonds, alkyl, and Pi-alkyl interactions with distances of 1.8, 3.0, 2.7, 2.6, and 5.3Å, respectively (Figure 6A). The binding site for benzothiazole involved residues Lys72, Phe65, and Ile39, with hydrogen bonds, Pi-sulfur, and Pi-alkyl interactions at distances of 1.9, 4.6, and 5.2Å (Figure 6B). For CYP6AEL1, the predicted binding site for ethyl formate involved residues Val190, Val189, and Val141, engaging in alkyl and carbon-hydrogen bonds at distances of 4.7, 2.5, and 4.4Å (Figure 6C). The binding site for benzothiazole included Lys72, with Pi-cation and Pi-alkyl interactions at distances of 3.8 and 4.3Å (Figure 6D). The binding energies for the CYP6SZ3-ethyl formate, CYP6SZ3-benzothiazole, CYP6AEL1-ethyl formate, and CYP6AEL1-benzothiazole were −94.68, −26.89, −70.85, and −26.88 kcal/mol, respectively.

## 4 Discussion

In the present study, two *CYP6* genes, *CYP6SZ3* and *CYP6AEL1*, were identified which belong to separate branches of the CYP6 family. The deduced amino acid sequences of two CYP6s contain five conserved motifs: WxxxR, AGxE/DTT/S, ExLR, PxxFxPE/DRF, and FxxGxRxCxG/A (Liang et al., 2015; Xu et al., 2024; Li et al., 2024). Many members of CYP6 family are known to play roles in xenobiotic metabolism, aiding in detoxification (Katsavou et al., 2022), such as *CYP6M2* and *CYP6P3* in *Anopheles gambiae* (Djouaka et al., 2008), and *CYP6ER1* and *CYP6AY1* in *Nilaparvata lugens* (Zhang et al., 2016). Two *CYP6* genes are also typically expressed differentially across various developmental stages and tissues of *L. serricorne*. *CYP6SZ3* and *CYP6AEL1* showed higher expression levels in the larval stage, which is consistent with the expression patterns of other *CYP* genes like *CYP321A8*, *CYP321A9*, and *CYP321B1* in *Spodoptera frugiperda* (Zhang et al., 2020), and *CYP341B21*, *CYP341B37* in *Spodoptera litura* (Li et al., 2024). On the other hand, lower expression levels were observed in the prepupal and adult stages, similar to *CYP353A1* in *T*. *castaneum* (Zhu et al., 2013). The tissue-specific expression patterns of these genes suggest a specialized role in insect metabolism. For example, CYP6E12 is highly expressed in the fat body of *H*. *armigera* (Wang et al., 2018), while *CYP6BQ9* is predominantly expressed in the central nervous system and less so in the fat body of *T. castaneum* (Zhu et al., 2010). In this study, *CYP6SZ3* and *CYP6AEL1* exhibited high expression levels in the foregut, midgut, and fat body, tissues critical for detoxification in insects. The midgut and fat body play key roles in neutralizing allelochemicals (Skowronek et al., 2021; Zhu et al., 2011; Zeng et al., 2024), and the high expression levels in these tissues suggest that *CYP6SZ3* and *CYP6AEL1* are important for detoxifying insecticides.

Increased metabolic detoxification is often associated with the overexpression or induction of CYPs (Zhu et al., 2010; Wang et al., 2020). Therefore, this study focused on the role of the significantly induced CYP6 family in detoxifying fumigants. The results showed that the expression of *CYP6SZ3* and *CYP6AEL1* was significantly induced after exposure to LC_30_ doses of ethyl formate and benzothiazole, indicating their potential role in fumigant detoxification. However, no significant change was observed at the LC_50_ concentration of ethyl formate, possibly because higher doses might not allow enough time for the upregulation of protective CYPs before irreversible toxicity sets in. This trend is similar to what has been observed in *N*. *lugens*, where overexpression of *CYP6ER1* was limited under higher doses of imidacloprid (Bass et al., 2011). At higher doses, the toxic effects of ethyl formate on *L. serricorne* larvae may suppress detoxification functions or trigger apoptosis. Another possibility is that other detoxification genes, rather than *CYP6* genes, may play a dominant role when induced by high doses of ethyl formate, as seen with the co-involvement of *CYP6* and *CYP4* in imidacloprid metabolism in *N. lugens* (Zhang et al., 2016). Further investigation is needed to test these hypotheses.

Although *CYP6SZ3* and *CYP6AEL1* are potentially involved in the detoxification of ethyl formate and benzothiazole, their exact roles remain unclear. To explore their contribution to insecticide sensitivity, RNAi-based reverse genetics approaches were used (Li et al., 2024; Zhang Z. X. et al., 2023). After RNAi treatment, the expression of *CYP6SZ3* and *CYP6AEL1* was significantly reduced, resulting in increased mortality in *L. serricorne* when exposed to ethyl formate or benzothiazole. These results suggest that downregulation of these genes increases susceptibility to fumigants. Several CYP6 family genes from Coleopteran insects have been functionally analyzed for their roles in insecticide detoxification. For example, silencing of *CYP6BJa/b* and *CYP6BJ1v1* in *Leptinotarsa decemlineata* increases susceptibility to neonicotinoids (Kalsi and Palli, 2017). Similarly, dsRNA-mediated knockdown of *CYP6BQ9* in *T*. *castaneum* has been shown to enhance susceptibility to deltamethrin (Zhu et al., 2010), a finding further supported by *in vitro* detoxification studies. Suppressing *CYP6BQ11* expression has been found to increase susceptibility of *T*. *castaneum* to dichlorvos and carbofuran (Xiong et al., 2019). Based on these findings, it is likely that *CYP6SZ3* and *CYP6AEL1* contribute to enhanced tolerance against ethyl formate and benzothiazole. However, it is necessary to make clear whether *CYP6SZ3* and *CYP6AEL1* have the function of metabolizing ethyl formate and benzothiazole, the subsequent fumigant metabolism assays is needed.

Molecular modeling and docking analyses were performed to investigate the mechanism by which these two CYP6 proteins interact with fumigants. Molecular docking is a powerful tool for predicting interactions between insecticides and detoxification enzymes (Xiao and Lu, 2022; Tang et al., 2024). In this study, human CYP3A4 and CYP3A5 were used as templates to build models for CYP6SZ3 and CYP6AEL1. The docking results indicated that both ethyl formate and benzothiazole bind strongly to CYP6SZ3 and CYP6AEL1. Several key residues, mostly hydrophobic amino acids such as Phe, Val, Leu, and Ile, appear to be involved in the binding (Zhu et al., 2010; Zhu et al., 2013). The binding free energies for CYP6SZ3 and CYP6AEL1 with the fumigants ranged from −26.88 to −94.68 kcal mol^−1^, indicating strong binding affinities. These findings indicate that these genes might play essential roles in metabolizing the ethyl formate and benzothiazole, although further research involving heterologous expression and metabolic assays is required to confirm their metabolic capacity.

## Data Availability

The datasets presented in this study can be found in online repositories. The names of the repository/repositories and accession number(s) can be found in the article/Supplementary Material.
