# Supplementary material for: Knockdown of CYP6SZ3 and CYP6AEL1 genes increases the susceptibility of Lasioderma serricorne to ethyl formate and benzothiazole
Source: Front Physiol. 2024 Nov 20;15:1503953. doi: 10.3389/fphys.2024.1503953 (PMC11615064; doi:10.3389/fphys.2024.1503953)
Supplement: Supplementary file 1 [file Table1.docx]

**Table S1. Primers used in this study.**

| **Application Primers** | **Gene Name** | **Forward Primer (5'-3')** | **Reverse Primer (5'-3')** |
| --- | --- | --- | --- |
| ORF confirmation | *CYP6SZ3* | CGAAGGATTGAAATGGAGAA | GGCTGTTCTGGTTCCGCTCT |
|  | *CYP6AEL1* | CCACTTCAAACGTCTCGGCA | CAACTTCCAATAGTACTTGC |
| qPCR analysis | *CYP6SZ3* | AGAGGACCGGCAGCTAAATT | ATGGTGAATCTGGCCGAAGT |
|  | *CYP6AEL1* | TGATCCTCGACCCAGATGTG | ACATCCCCTTCATCTTCCCG |
| dsRNA synthesis | *CYP6SZ3* | **TAATACGACTCACTATAGGG**CAGACAAGAACTCAAATCAC | **TAATACGACTCACTATAGGG**CTCCCAGATACACTTATCGC |
|  | *CYP6AEL1* | **TAATACGACTCACTATAGGG**AGTCCTACATTCACTTCCGG | **TAATACGACTCACTATAGGG**TCTTTATGTTGCGCCAACTC |
|  | *GFP* | **TAATACGACTCACTATAGGG**CAGTTCTTGTTGAATTAGAT | **TAATACGACTCACTATAGGG**AATGTTACCATCTTCTTTAA |

*The sequence in bold at the 5’end of the primer is the T7 promoter sequence.
